# Marketing and infant and young child feeding in rapidly evolving food environments

**DOI:** 10.1111/mcn.12810

**Published:** 2019-06-21

**Authors:** Elizabeth Zehner, Mary Champeny, Sandra L. Huffman

**Affiliations:** ^1^ Helen Keller International New York New York USA; ^2^ Consultant to Helen Keller International New York New York USA

## 1 INTRODUCTION

Optimal infant and young child feeding practices, including exclusive breastfeeding for the first 6 months of life followed by the introduction of nutritious complementary foods and the continuation of breastfeeding up to 2 years of age or beyond, are vital for children's growth and development (Victora et al., 2015, 2016; World Health Organization [WHO], 2003). If scaled up to near universal levels, optimal breastfeeding could save 823,000 child lives each year across 75 low and middle income countries (LMIC) (Victora et al., 2016) and has the potential to reduce long‐term morbidities (Kørvel‐Hanquist, Djurhuus, & Homøe, 2017; Morris, 2018; Wang, Collins, Ratliff, Xie, & Wang, 2017). However, only 28% of infants born in LMIC begin breastfeeding within an hour of birth, and in most countries, rates of exclusive breastfeeding to 6 months of age are below 50% (Victora et al., 2016). Global sales volume of breastmilk substitutes (BMS) for children 0–6 months increased by 54.9% from 2005 to 2017, while sales grew for all BMS products in all regions except North America (Development Initiatives, 2018). Across the world's least developed countries, just 12% of children aged 6–23 months achieve a minimum acceptable diet apart from breastmilk, indicating that complementary foods are often lacking in frequency, diversity, and age appropriateness (United Nations Children's Fund [UNICEF], 2017). Concurrently, consumption of energy‐dense but nutrient‐poor snack foods is increasingly prevalent during this nutritionally vital complementary feeding period (Huffman, Piwoz, Vosti, & Dewey, 2014). Across the world, per capita sales of packaged foods increased from 67.7 kg in 2005 to 76.9 in 2016, with significant growth occurring in LMIC economies in Asia and Africa (Development Initiatives, 2018). The majority of the world's low birthweight, stunting, and wasting, as well as child mortality, appear in LMIC (UNICEF, 2016).

From 2013 to 2016, Helen Keller International's Assessment and Research on Child Feeding (ARCH) Project conducted research on promotion and consumption of commercial foods fed to children less than 24 months of age in the largest urban areas of four countries with high burdens of malnutrition. Studies were conducted in Phnom Penh, Cambodia; Kathmandu Valley, Nepal; Dakar, Senegal; and Dar es Salaam, Tanzania, in conjunction with Ministries of Health in each site (Zehner & Champeny, 2016).

This 2019 *Maternal & Child Nutrition* supplement presents the continuation of research in Cambodia, Nepal, and Senegal, as well as a broadening of its scope in a new study site, Bandung City, Indonesia, and among a new age group: children 24–35 months of age. The nine articles presented here provide new insights into the promotion which takes place for these products in urban LMIC study sites, exploration of the nutrient content and fortification of commercial products consumed by young children, as well as new data on consumption of commercial foods by infants and young children and drivers of food choice for commercial foods by caregivers.

## 2 PROTECTION OF BREASTFEEDING: THE MARKETING REALITY AND COUNTRY‐LEVEL EXPERIENCES IN POLICY IMPLEMENTATION

Over the last four decades, global policies and guidance have been developed to safeguard infant and young child nutrition in the context of changing food environments. In 1981, the World Health Assembly adopted the International Code of Marketing of Breast‐milk Substitutes, which proscribes the promotion of BMS, as well as other food and beverage products when marketed or otherwise represented to be suitable as a partial or total replacement of breastmilk. However, these provisions have been inconsistently implemented and enforced (Mcfadden, Kenney‐muir, Whitford, & Renfrew, 2015; Robinson, Buccini, Curry, & Perez‐Escamilla, 2019; WHO, 2018), leaving caregivers exposed to commercial marketing for BMS through many promotional avenues and indicating a need for more robust monitoring systems (Hernández‐Cordero et al., 2019). Recent analysis suggests that full implementation of the Code is necessary but not sufficient to significantly improve breastfeeding outcomes in the absence of other enabling factors (Robinson et al., 2019).

Television advertising of BMS and commercially produced complementary foods was monitored in Phnom Penh and Dakar, two cities where regulatory environments for these food products differ, despite national adoption of the International Code of Marketing of Breast‐milk Substitutes. While television advertising for infant and young child feeding products are not specifically addressed in Senegalese regulations, advertisements can be aired in Cambodia with Ministry of Health approval. Descriptive analysis of this advertising is presented in Champeny, Hou, et al. (2019). While no advertisements in either site were observed for BMS for infants younger than 6 months, advertisements were observed for BMS for older infants and young children, and in most cases, recommended ages for use were not specified. Nutrition and health claims were commonly seen in both BMS and commercially produced complementary food advertisements.

In Champeny, Pries, et al. (2019), mothers were interviewed about their use of BMS prior to discharge after delivery in Phnom Penh and Kathmandu Valley maternal health facilities. In both sites, over half of infants were fed BMS while in the health facility. Authors explored factors associated with introduction of BMS and found that recommendations from health professionals to mothers to feed BMS were significantly related to use in both sites. In both Phnom Penh and Kathmandu Valley, national legislation restricts BMS promotion within health facilities.

Globally, the market for BMS continues to grow, with sales in 2014 reaching over $44 billion USD and projected to reach $70.6 billion USD by 2019 (Rollins et al., 2016). Since the adoption of the International Code of Marketing of Breast‐milk Substitutes, manufacturers have created new categories of BMS in addition to the traditional “infant formula,” including “follow‐on formulas” and “growing‐up milks,” with packaging and labelling similar to infant formula and which are widely promoted (Pereira et al., 2016). This marketing practice creates confusion among consumers (Berry, Jones, & Iverson, 2012; Cattaneo et al., 2015) and may lead to misuse of these milks and related products with potentially dangerous consequences.

In response to the changing commercial food product landscape and repeated calls for additional clarification, the World Health Assembly adopted a resolution (WHA 69.9) in 2016 calling on countries to implement the World Health Organization's *Guidance on Ending the Inappropriate Promotion of Foods for Infants and Young Children* (WHO, 2016). The guidance encourages Member States to strengthen national policies that protect children under 3 years of age from marketing practices that could be detrimental to their health (WHO; UNICEF; Save the Children; IBFAN; Helen Keller International, 2016). Yet full implementation of the Code and subsequent relevant WHA resolutions including WHA 69.9 remains a challenge (WHO, UNICEF, & IBFAN, 2016; WHO, 2018).

Hou et al. (2019) report on the development and piloting of a monitoring and enforcement system in four urban areas of Cambodia to assess compliance with national legislation restricting the marketing of infant and young child feeding products. Assessment of point‐of‐sale promotions, labels/packaging of products, and promotional materials and activities within health facilities was conducted by staff from three departments of two different government ministries. Multiple violations were found; authors report that point‐of‐sale violations (86.0%) were more prevalent than label (53.0%) or health facility violations (40.5%). However, no reports were submitted on these violations to the Executive Working Group tasked with enforcement of the legislation in 2014. This paper illustrates challenges involved in scaling‐up monitoring and enforcement mechanisms. It also highlights the need for dedicated resources to adequately conduct monitoring. Activities within monitoring systems need to be assessed to ensure they are operating as expected and that operations are evaluated for appropriateness. Systems must be revised and evolve as needed to facilitate sustainability. It should be noted that since the submission of this manuscript for publication, citations for non‐compliant companies have been issued by the Executive Working Group in 2019.

## 3 THE POINT‐OF‐SALE LANDSCAPE: AN IN‐DEPTH ASSESSMENT OF MARKETING OF COMMERCIAL FOODS IN INDONESIA

In addition to BMS, other categories of commercial food products can pose challenges for optimal young child feeding. Around the age of 6 months, an infant's nutritional needs increase beyond what exclusive breastfeeding can provide (WHO, 2003). This period commonly corresponds to growth faltering in young children and is an important focus area for preventing future childhood malnutrition (de Onis & Branca, 2016). The introduction of safe, adequate, and nutrient‐dense complementary foods is necessary at this point, while breastfeeding continues. While WHO recommends the use of low‐cost fortified complementary food products as needed, these products must be promoted in a way that protects breastfeeding and the consumption of diverse diets based on locally available foods. Promotion of complementary foods and beverages before 6 months of age is associated with earlier cessation of exclusive breastfeeding (WHO, 2016), increasing the risks of infection and other morbidities.

Commercial complementary foods vary widely in nutritional quality, with some providing micronutrients that are missing in the typical diets of young children, while others are of concern because they have high levels of added salt or sugar or contain industrially produced trans‐fatty acids. The WHO *Guidance on Ending Inappropriate Promotion of Foods for Infants and Young Children* considers promotion of products containing high levels of sugar or salt or contributing to childhood obesity and non‐communicable diseases to be inappropriate (WHO, 2017). Implementation of the *Guidance* around complementary foods poses many challenges, including the difficulties inherent in identifying nutritious commercial complementary foods and distinguishing them from poor quality complementary food products that should not be promoted. In addition, commercially produced snack foods/beverages, typically high in added salt or sugar, are increasingly available in LMIC. Though these products are not formulated for infants and young children, they are increasingly fed to this young age group with limited to no restrictions on child or caregiver‐targeted marketing of these products.

Acknowledging the range of commercial products available and promoted to caregivers of young children, Hadihardjono et al. (2019) report on promotions of BMS, commercially produced complementary foods, and commercial snack foods and beverages observed at points‐of‐sale in Bandung City, Indonesia. Nearly all of the 43 sampled stores sold complementary foods (*n* = 42) and selected commercial snacks (*n* = 41). There were 147 different BMS products identified in 28 stores. Of these 147 products, 63.3% were found promoted in stores. Over 80% of stores carrying BMS and commercially produced complementary foods had promotions for these products, and nearly all stores had promotions for selected commercial snacks. Promotions for BMS through 1 year of age are restricted by Indonesian legislation. Only 4.0% of the total number of BMS promotions included products for infants less than 12 months of age, but they were found in 42.9% of stores selling BMS. Almost all BMS promotions included a BMS for children 12 months and over (“growing‐up milk”) and half included a joint promotion with a commercial snack product.

## 4 UNDERSTANDING NUTRIENT CONTENT OF COMMERCIAL FOODS COMMONLY CONSUMED BY INFANTS AND YOUNG CHILDREN: EVIDENCE FROM INDONESIA

The use of fortified infant foods has been included in strategies to improve child nutrition after age 6 months since they can improve the intake of iron, zinc, and other nutrients often lacking in diets of young children (Campos Ponce, Polman, Wieringa, & Doak, 2018; Leyvraz et al., 2016; Lutter et al., 2011; Plessow, Arora, Brunner, & Wieser, 2016; Wieser et al., 2018; WHO, 2003). They have been shown to lead to enhanced growth (Diana et al., 2017; Ten Year Strategy to Reduce Vitamin and Mineral Deficiencies Maternal Infant and Young Child Working Group Formulation Subgroup, 2009) and improved iron status (Faber, Kvalsvig, Lombard, & Benadé, 2005; Lutter, 2008). However, studies assessing forms of micronutrients used or amounts provided in products sold on the market have been limited (Dimaria et al., 2018; Masters, Nene, & Bell, 2017). Without fortification requirements specifying minimum levels and forms of micronutrients, or labels which indicate nutrient levels per serving, it can be difficult to determine if the amount provided is appropriate or will have the desired effect on micronutrient status. Inappropriate promotion of commercial complementary foods and beverages can confuse caregivers about the nutritional qualities of these foods and beverages and about their age appropriate and safe use, especially in the absence of clear information on the content of iron and other nutrients.

The iron content of 217 complementary foods sold in stores included in the Bandung City study (Hadihardjono et al., 2019) was assessed by Dreyfuss et al. (2019) through a review of labels on 95 infant cereals, 71 snacks/finger foods (biscuits/cookies, puffs, and noodles/crackers), 35 purees, and 16 other foods. The recommended age of use on product labels varied by the type of product; most (81%) cereals were recommended for use beginning at 6 months of age, while 60% of snacks/finger food, 70% of pureed food, and 100% of mixed meals and pudding were recommended for use starting at 1 year of age. About two‐thirds (69.6%) of labels stated that the products contained iron, but 58% indicated they were iron fortified. Only one fifth of fortified products indicated the type of iron used, but all of those included a form of iron recommended by WHO for fortifying complementary foods. Infant cereal was more likely to be fortified and to contain more iron compared to snacks/finger food and purees.

## 5 EXPLORING WHAT IS KNOWN ABOUT UNHEALTHY DIETS AMONG YOUNG CHILDREN

Foods specifically designed for infants and young children are not the only commercial products fed to young children. Consumption of commercially produced snack foods, often high in sugar or salt, is a growing trend across many countries (Huffman et al., 2014). The 2015 Lancet series on obesity (Lobstein et al., 2015) noted that promoting energy‐rich and nutrient‐poor products encourages rapid weight gain in early childhood and exacerbates risk factors for chronic disease in children. The extent to which such foods are used for young child feeding in LMIC and the influence of these shifting diet patterns on young child nutrition is an emerging topic for exploration.

Pries, Filteau, and Ferguson (2019) conducted a systematic review of the contribution of snack food and sugar‐sweetened beverages to total energy intakes of children less than 2 years of age in LMIC and the relationship with nutritional outcomes. Among nine studies detailing contribution to energy intakes among children under 24 months in LMIC, total energy intakes from snack foods/beverages ranged from 13% in Peru to 38% in Cambodia. An additional four papers assessed the relationship between consumption of these foods and nutritional outcomes, primarily ponderal growth, but varying use of snack food/beverage definitions and measurement of consumption limited study comparison. No studies investigated the relationship between unhealthy snack food/beverage consumption and dietary adequacy, and only one explored micronutrient status but only considering anaemia. This review highlighted a gap in knowledge on the role of these foods in young children's diets and nutritional outcomes in LMIC.

## 6 GENERATING NEW DATA ON COMMERCIAL SNACK FOOD CONSUMPTION: PREVALENCE OF CONSUMPTION IN INDONESIA AND DRIVERS OF CHOICE FOR SNACK FOODS IN NEPAL

Given that this systematic review documented a significant need for further research around the topic of snack food and beverage consumption among young children, further exploration around the prevalence of consumption and contribution of energy‐dense, nutrient‐poor snack foods to overall diets and reasons why caregivers opt to use these foods for young child feeding was conducted.

Green et al. (2019) report on consumption patterns among 495 children 6–35 months of age interviewed as part of a cross‐sectional survey in Bandung City. Consumption of commercial infant and young child feeding products was prevalent, with 49.5% and 37.4% of children having consumed a BMS or commercially produced complementary food in the previous day. Nearly half (46.5%) of children 6–11 months consumed a commercial snack food compared to almost all (91.9%) children 30–35 months. Consumption of any sugar‐sweetened beverages increased from 2.0% to 60.6% between the youngest and oldest age groups. Among all children, sweet biscuits (56.8%) and savoury snacks (47.3%) were the most commonly consumed foods, and sweetened milk (33.3%) was the most commonly consumed sugar‐sweetened beverage. Children of less educated mothers were more likely to have consumed a snack food compared to mothers with post‐secondary level of education.

Pries, Sharma, et al. (2019) report on nutrient profiles of snack foods and beverages consumed by young children in Kathmandu Valley, Nepal, and assessed the proportion of total energy intake from foods/liquids other than breastmilk contributed by snack foods and sugar‐sweetened beverages. On average, unhealthy snack foods and beverages contributed 24.5% of total energy intake from foods/liquids other than breastmilk among children 12–23 months of age, and nearly all unhealthy snack foods and beverages were commercially produced. These products also contributed 31.1% of total sugar and 44.9% of total sodium intake. Biscuits (10.8% total energy intake), candy/chocolate (3.5%), and savoury snacks (3.4%) provided the largest percent of total energy intake from snacks. Children in the poorest households were more likely to be high consumers of commercial snacks and sugar‐sweetened beverages, while children of caregivers who had higher educational levels were less likely to be high consumers. Authors suggest that to reduce unhealthy snack food and beverage consumption, interventions should seek to further understand drivers of feeding practices, target disadvantaged populations, and ensure that caregivers are aware of the nutritional quality of food products for their children.

Sharma et al. (2019) assessed caregivers' perceptions of commercial snack foods/beverages and factors influencing their use for young child feeding. Seven focus group discussions and a survey of 745 primary caregivers of children 12–23 months were conducted in Kathmandu Valley (Pries, Sharma, et al., 2019). Authors report almost all children (98.4%) had consumed a snack food (such as biscuits/cookies, candy, potato chips, and cheeseballs) or sugar‐sweetened beverage in the week prior to interview; biscuits, candy/chocolates, savoury snacks, and instant noodles were the most commonly consumed, by 92.1%, 82.8%, 66.0%, and 59.2% of children. Biscuits were often mixed with milk and fed as a meal. Most commercial snack foods were perceived by caregivers to be unhealthy for young children and not trusted, but caregivers reported feeding commercial snacks and sugar‐sweetened beverages because the child liked them and because they were convenient (easy to prepare or ready‐to‐eat or easy to feed to the child).

## 7 CONCLUSIONS

This supplement extends previous findings on promotion and consumption of commercial foods and beverages among infants and young children and illustrates the widespread promotion and high rates of consumption in LMIC. Evidence in this supplement underscores the need to adopt, monitor, and enforce legislation prohibiting BMS promotion, as well as implement laws and policies to prevent inappropriate promotion of commercially produced complementary foods. Ensuring consistency with global standards is necessary to protect, promote, and support optimal infant and young child feeding.

Understanding the consumption patterns and the promotional messages mothers receive from the commercial sector about infant and young child feeding is also needed so that programmes can reinforce positive messages about optimal infant feeding and tailor messages and interventions to address the promotion of BMS, inappropriate promotion of commercially produced complementary foods, and consumption of commercial snack products among young children. As noted by Pries, Sharma, et al. (2019), further research is needed to understand how consumption of energy‐dense, nutrient‐poor foods and beverages influences undernutrition and overnutrition among young children during the complementary feeding period. The first 3 years of life present a critical window of opportunity to protect and promote good nutrition and healthy dietary patterns. With wide availability of highly processed foods and beverages and growing evidence that children are consuming commercial snack products, attention must be focused on increasing consumption of nutrient‐rich, locally available foods during the complementary feeding period and developing strategies to limit consumption of unhealthy foods and beverages.
